# Micronutrient supplementation during pregnancy: evidence, indications, and safety - Number 14 – 2026

**DOI:** 10.61622/rbgo/2026FPS14

**Published:** 2026-09-14

**Authors:** Lílian de Paiva Rodrigues Hsu, Eliana Martorano Amaral, Adriana Gomes Luz, Eura Martins Lage, Mônica Lopez Vazquéz, Joeline Maria Cleto Cerqueira

**Affiliations:** 1 Santa Casa de São Paulo Faculdade de Ciências Médicas São Paulo SP Brazil Faculdade de Ciências Médicas, Santa Casa de São Paulo, São Paulo, SP, Brazil.; 2 Universidade Estadual de Campinas Faculdade de Ciências Médicas Campinas SP Brazil Faculdade de Ciências Médicas, Universidade Estadual de Campinas, Campinas, SP, Brazil.; 3 Universidade Estadual de Campinas Faculdade de Ciências Médicas Campinas SP Brazil Faculdade de Ciências Médicas, Universidade Estadual de Campinas, Campinas, SP, Brazil; 4 Universidade Federal de Minas Gerais Faculdade de Medicina Belo Horizonte MG Brazil Faculdade de Medicina, Universidade Federal de Minas Gerais, Belo Horizonte, MG, Brazil.; 5 Santa Casa de São Paulo Faculdade de Ciências Médicas São Paulo SP Brazil Faculdade de Ciências Médicas, Santa Casa de São Paulo, São Paulo, SP, Brazil.; 6 Universidade Estadual do Piauí Teresina PI Brazil Universidade Estadual do Piauí, Teresina, PI, Brazil.

## Abstract

•Structured nutritional assessment during prenatal care is essential for rationalizing therapy and promoting equity in healthcare.

•Decisions regarding micronutrient supplementation during pregnancy must distinguish between interventions offering population-level benefits (for populations with low micronutrient intake) and strategies based on individual risk.

•Folic acid and iron remain the supplements most strongly supported by robust and consistent evidence.

•Calcium supplementation reduces the risk of preeclampsia in populations with low dietary intake and is indicated for specific subgroups or populations with documented deficiency.

•The indiscriminate prescription of multivitamins lacks consistent support in the literature and leads to unnecessary, excessive medicalization during prenatal care.

•Evidence regarding universal supplementation with vitamin D, docosahexaeonic acid (DHA/omega-3), vitamin B12, and iodine is inconsistent. Such supplementation is indicated for specific subgroups or populations with documented deficiency.

•Tailoring supplementation to the individual represents an advance in evidence-based practice without replacing interventions of proven population-level benefit.

## Recommendations

- Ensure universal folic acid supplementation during the periconceptional period and the first trimester given its efficacy in preventing neural tube defects (NTDs).
- Ensure universal iron supplementation during pregnancy, considering the high prevalence of maternal anemia and its association with preterm birth, low birth weight, and maternal morbidity and mortality.
- Recommend calcium supplementation for pregnant women with insufficient dietary intake, prioritizing contexts of nutritional vulnerability.
- Adopt an individualized approach to vitamin D supplementation based on risk factors for hypovitaminosis or documented deficiency.
- Nutritional risk groups such as vegetarians, vegans, and women who have undergone bariatric surgery or have malabsorption or a confirmed deficiency, require a specific, individualized approach.
- Consider DHA (omega-3) supplementation for pregnant women with low intake of marine fish or an increased risk of preterm birth, acknowledging the moderate benefit.
- Discourage the indiscriminate prescription of multivitamins without a sound clinical indication, reinforcing therapeutic rationale during prenatal care.

## Background

Ensuring adequate maternal nutritional status is a cornerstone of prenatal care, directly impacting fetal development, maternal physiological adaptation, and the risk of adverse obstetric outcomes.

Interventions such as folic acid and iron supplementation are supported by robust evidence of population-level benefits. In Brazil, public health policies, including mandatory flour fortification and universal salt iodization have substantially shifted the epidemiological profile of nutritional deficiencies.

The higher prevalence of obesity, bariatric surgery, and restrictive diets among pregnant women underscores the need for an individualized approach. Rational supplementation, grounded in evidence and the epidemiological context, is crucial for preventing adverse outcomes, particularly in vulnerable populations.

## Folic acid (vitamin B9, methylfolate)

### What is the impact during the periconceptional period?

Folic acid, the synthetic form of vitamin B9, is an essential cofactor for DNA synthesis and cellular methylation, which are critical processes during organogenesis. The variable bioavailability and thermal instability of dietary folate justify pharmacological supplementation during the periconceptional period.^(1)^

Maternal folate deficiency is consistently associated with neural tube defects (NTDs), and an increased risk of preterm birth and low birth weight. Periconceptional supplementation is one of the preventive interventions with the highest level of evidence, significantly reducing the incidence of NTDs.^(1)^

### What is the importance of 5-methyltetrahydrofolate (5-MTHF)?

5-MTHF, the biologically active form of folate, plays a direct role in homocysteine metabolism and erythropoiesis. It is indicated for women with alterations in folic acid metabolism, as it improves folate levels, reduces homocysteine, and prevents the accumulation of unmetabolized folic acid. Recent studies suggest a possible additional benefit regarding endothelial function and a reduction in gestational hypertension.^(1)^

### What is the recommended dose?

For pregnant women at average risk, a dose of 400 mcg/day is recommended, starting at least 30 days before conception and continuing through the end of the first trimester.

For high-risk women such as those with a history of NTDs (personal or family), use of anticonvulsants, genetic polymorphism (MTHFR C-677T), diabetes mellitus, obesity, or bariatric surgery, a dose of 4–5 mg/day is recommended for the same period.^(1)^

## Iron

### What is the importance of iron during pregnancy?

Iron is essential for erythropoiesis and oxygen transport.^(2)^ Demand increases progressively during pregnancy due to the expansion of plasma volume and red blood cell mass, as well as fetal growth, often rendering dietary intake insufficient. Iron-deficiency anemia affects more than one-third of pregnant women worldwide and is associated with preterm birth, low birth weight, increased maternal morbidity and mortality, and adverse effects on child development.^(3)^

### Should prophylactic supplementation be universal?

The International Federation of Gynecology and Obstetrics (FIGO)^(4)^ recommends that all pregnant women receive daily iron supplementation, especially in settings where the prevalence of anemia reaches or exceeds 40%. In Brazil, the Brazilian Federation of Gynecology and Obstetrics Associations (Febrasgo)^(4)^ and the Ministry of Health ^(5)^ advise daily oral supplementation with 30 to 60 mg of elemental iron for all pregnant women.

### What is the best strategy for oral administration?

Ferrous sulfate, the most widely used salt, is best absorbed on an empty stomach taken with vitamin C. Concurrent administration with calcium, milk, tea, coffee, antacids, H2 blockers, and proton pump inhibitors should be avoided. Alternate-day dosing may increase absorption and reduce gastrointestinal side effects.^(6)^

### When is intravenous iron indicated?

It is indicated in cases of intolerance to or failure of oral iron therapy, moderate to severe anemia requiring a rapid response, or malabsorption conditions. Although it raises hemoglobin levels more quickly, meta-analyses have not demonstrated a significant difference in neonatal outcomes, such as preterm birth or mortality, though they have shown a slight increase in birth weight and higher umbilical cord ferritin levels.^(7)^

## Vitamin D

### What is the role of vitamin D in pregnancy?

Vitamin D acts as a hormone regulating calcium and phosphorus homeostasis, bone mineralization, immune modulation, and placental function. It contributes to fetal skeletal development, maternal-fetal immune tolerance, and placental function, including processes such as trophoblastic invasion and angiogenesis.^(8)^

Although there is no absolute consensus regarding the ideal cutoff point, values between 20 and 30 ng/mL are frequently considered adequate.^(9)^

### Should vitamin D supplementation be recommended?

Potential benefits of vitamin D supplementation include improved outcomes regarding preeclampsia (at doses up to 2,000 IU/day) and gestational diabetes (at doses exceeding 2,000 IU/day).^(10)^ A systematic review^(11)^ highlighted uncertainty regarding high-quality evidence due to limitations in the studies evaluated. Supplementation is generally viewed as a safe, low-risk intervention and is recommended in cases of deficiency.

The World Health Organization (WHO) recommends universal supplementation only for pregnant women with confirmed deficiency or limited sun exposure, while advising the promotion of a balanced diet and adequate sun exposure.^(12)^ Some international societies advocate for routine supplementation, considering the high global prevalence of hypovitaminosis D, the safety profile of moderate doses, and the potential impact on pregnancy outcomes.^(12-14)^

### Is vitamin D deficiency prevalent among Brazilian pregnant women?

A deficiency prevalence of 30% and an insufficiency prevalence exceeding 59% were observed, indicating national epidemiological significance.^(15)^

### What is a safe dose?

Oral cholecalciferol (vitamin D3) has a favorable safety profile. Doses between 800 and 2,000 IU/day are considered safe. The Endocrine Society supports routine supplementation of up to approximately 2,500 IU/day,^(16)^ with an upper limit of 4,000 IU/day considered tolerable.^(17)^

## Calcium

### Does calcium supplementation reduce pregnancy complications?

The meta-analysis by Hofmeyr et al. (2018)^(21)^, which concluded that calcium supplementation (≥1 g/day) significantly reduces the risk of pre-eclampsia, preterm birth, and severe maternal complications, with a more pronounced protective effect in populations with low dietary calcium intake, was recently re-evaluated.

### What is the current landscape of scientific evidence?

Current recommendations are based on a meta-analysis^(21)^ concluding that calcium supplementation (≥1 g/day) significantly reduces the risk of preeclampsia, preterm birth, and severe maternal complications, with a pronounced protective effect in populations with low dietary intake.

A critical update^(24)^ concluded that the evidence of benefit weakens substantially when the analysis is restricted to high-quality clinical trials, no longer robustly supporting the prevention of preeclampsia through supplementation.

Supplementation remains a vital public health strategy in contexts of low dietary intake and high clinical risk, pending further large-scale studies for definitive conclusions.

### What is the current recommendation?

The Brazilian Network for Studies on Hypertension in Pregnancy (Portuguese acronym: RBEHG) and Febrasgo's National Specialized Commissions (CNEs) on Hypertension in Pregnancy and Prenatal Care do not recommend the use of calcium for the prevention of preeclampsia in light of current evidence.

The WHO^(23)^ recommends 1.5–2 g/day for pregnant women with low calcium intake. The Ministry of Health^(24)^ recommends 1,000 mg (or 1 g) of calcium per day, from the 12 week of pregnancy until delivery.

### Are there risks associated with calcium supplementation?

Calcium supplementation is not associated with a significant increase in maternal or neonatal adverse events.^(23)^

## Vitamin B12

### What is the role of vitamin B12 in pregnancy?

Vitamin B12 (cobalamin) plays a role in DNA synthesis, erythropoiesis, and homocysteine metabolism, working in conjunction with folate in methylation processes and fetal neurological development. Maternal deficiency can result in megaloblastic anemia, hyperhomocysteinemia, and potential repercussions for the child's growth and neurodevelopment.

### Does supplementation offer clinical benefits?

A meta-analysis of randomized clinical trials demonstrated that supplementation significantly reduces the risk of maternal deficiency (25.9% vs. 67.9%; relative risk: 0.38; 95% confidence interval: 0.28–0.51) compared to placebo,^(25)^ without showing significant differences in outcomes such as preterm birth, low birth weight, or child cognitive development. Although effective in correcting deficiency, the benefit for pregnant women without risk factors remains uncertain.^(26)^

### What is the recommended dose?

The recommended daily intake for pregnant women is 2.6 μg. Supplementation is indicated only in cases of confirmed deficiency. The route of administration is oral when associated with insufficient intake, and parenteral in cases of malabsorption.^(27)^

### Which pregnant women are at higher risk of deficiency?

Women who follow vegetarian or vegan diets, have undergone bariatric surgery, or have conditions that impair intestinal absorption.^(28)^

## Omega-3 (docosahexaenoic acid or DHA)

### What is the role of DHA during pregnancy?

Omega-3 polyunsaturated fatty acids include alpha-linolenic acid, eicosapentaenoic acid (EPA), and DHA. Docosahexaenoic acid is a structural component of neuronal membranes and the retina and plays a central role in fetal brain development, particularly during the third trimester, a period of intense incorporation into neural tissue. Active placental transfer supports the growth of the central nervous system.

### Does supplementation alter obstetric outcomes?

Adequate DHA levels are associated with higher birth weight and improved fetal growth.^(29)^ Studies show a reduced risk of preterm birth before 34 weeks and prematurity before 37 weeks in women with low omega-3 intake.^(30)^ Evidence regarding neurodevelopment and infant visual acuity is mixed and depends on the mother's baseline nutritional status.^(31)^

### What is the recommended intake?

Supplementation is most beneficial in contexts of low marine fish intake or a higher risk of prematurity. A daily intake of approximately 250 mg of omega-3 (EPA + DHA) is recommended, with an additional 200 mg of DHA during pregnancy.^(32)^ Supplementation should prioritize products certified for purity and tested for contaminants.

## Choline

### What is the role of choline during pregnancy?

Choline is involved in the synthesis of cell membrane phospholipids, acetylcholine production, homocysteine metabolism, and methyl group donation, playing a central role in methylation and epigenetic regulation. It contributes to fetal brain formation, placental function, and the programming of neurodevelopment.^(33)^

### Is maternal intake adequate, and what is the impact of deficiency?

Most pregnant women do not meet the recommended intake. In some populations, this figure may exceed 80%.^(34)^ The recommendation for pregnant women in Brazil is 450 mg/day.^(35)^ A meta-analysis reports up to a 2.36-fold increase in the risk of NTDs in contexts of low intake. Adequate maternal levels are associated with better fetal brain development and child neurodevelopment after birth.^(36)^

### When should supplementation be considered?

Supplementation may be considered in cases of inadequate dietary intake associated with low consumption of animal-based foods or restrictive diets. A systematic review and meta-analysis evaluated eight international nutritional guidelines regarding recommendations for pregnant women, highlighting the importance of choline for maternal health and fetal development, and linking its intake to a lower risk of obstetric complications.^(36)^

## Iodine

### Does routine iodine supplementation offer clinical benefits?

Available evidence is limited and heterogeneous. A systematic review found insufficient evidence regarding the benefits or risks of routine iodine supplementation during the preconception, pregnancy, and postpartum periods.^(37)^ Among pregnant women living in iodine-deficient regions, there is no significant impact on maternal outcomes or child neurodevelopment.^(38)^ In Brazil, the consumption of iodized salt contributes to reducing deficiency in the general population.

### What is the recommended daily intake?

The Institute of Medicine recommends 220 µg/day during pregnancy.^(39)^ The WHO recommends supplementation in populations with proven deficiency.^(40)^ The American Thyroid Association advises a total intake of approximately 250 μg/day for pregnant women, recommending supplements containing 150 μg of iodine.^(41)^

### Is there a risk associated with excessive intake?

Although uncommon, excess intake can cause maternal and fetal thyroid dysfunction. Cases of fetal goiter and congenital hypothyroidism have been described with maternal intake ranging from 2.3 to 12.5 mg/day.^(42)^ However, excessive intake is rare, occurring in less than 1% of pregnant women.^(43)^

## Magnesium

### What is the role of magnesium in pregnancy?

Magnesium acts as a cofactor in multiple enzymatic reactions, playing a role in neuromuscular excitability, vascular tone regulation, energy metabolism, and protein synthesis. It is involved in fetal tissue formation, steroidogenesis, and the modulation of uterine contractility.^(44)^

### Is magnesium deficiency associated with obstetric complications?

The association between low magnesium levels and an increased risk of preterm birth, preeclampsia, and miscarriage has not been confirmed in randomized clinical trials.^(45)^

### Is supplementation recommended?

Evidence is insufficient to recommend universal magnesium supplementation during pregnancy.^(46)^

## Final considerations

Micronutrient supplementation during pregnancy should be guided by scientific evidence regarding clinical benefit. Folic acid and iron remain universal interventions supported by their population-level impact. Other forms of supplementation should take into account risk factors and the epidemiological context. Rational prescribing practices prevent indiscriminate supplementation. Evidence-based practice requires a balance between effective prevention and the responsible use of therapeutic interventions. Table 1 outlines the micronutrients and relevant recommendations.

**Table 1:** Recommendations for micronutrient supplementation during pregnancy

Category	Micronutrient	Recommended Dosage
Universally Indicated	Folic Acid	400 mcg/day (0.4 mg) for low periconceptional risk and first trimester. For high risk (history of NTD, diabetes, obesity, or anticonvulsants), the dose is 4-5 mg/day.
	Elemental Iron	30 to 60 mg/day of elemental iron, with supplementation recommended to start between the 12 and 20 week and continue until 42 days postpartum.
To be individualized	Calcium	1,000 to 1,500 mg/day (or 1.5 to 2 g/day according to WHO in low-intake populations).
	Vitamin D	2,000 IU/day of cholecalciferol (vitamin D3), starting in the preconception period and continuing through lactation.
	Iodine	150 mcg/day (supplemental) in regions with diets known to be low in iodine. Target total intake of 220 to 250 μg/day.
	Vitamin B12	2.6 μg/day (pregnant women); mandatory for vegetarians, vegans, or post-bariatric surgery patients.
	Omega-3 (DHA)	200 mg/day of DHA additional to recommended dietary intake for neurodevelopment support and reduction of preterm birth.
	Choline	450 mg/day (ANVISA) to 480 mg/day (EFSA) to support fetal liver function and brain development when the diet is insufficient.
No Evidence for Universal Indication	Magnesium	Studies with 300 mg/day of magnesium citrate showed no reduction in adverse outcomes.

Source: Prepared by the authors

## Data Availability

research data are available in the article.

## National Commission Specialized in Prenatal Care of the Brazilian Federation of Gynecology and Obstetrics Associations (Febrasgo)

President:

Lilian De Paiva Rodrigues Hsu

Vice-president:

Eliana Martorano Amaral

Secretary:

Adriana Gomes Luz

Members:

Debora Farias Batista Leite
Eugenia Glaucy Moura Ferreira
Eura Martins Lage
Fernanda Garanhani De Castro Surita
Joeline Maria Cleto Cerqueira
Jorge Fonte De Rezende Filho
Julio Elito Junior
Monica Lopez Vazquez
Sigrid Maria Loureiro De Queiroz Cardoso
